# Effects of Calorie Restriction and IGF-1 Receptor Blockade on the Progression of 22Rv1 Prostate Cancer Xenografts

**DOI:** 10.3390/ijms140713782

**Published:** 2013-07-03

**Authors:** Colette Galet, Ashley Gray, Jonathan W. Said, Brandon Castor, Junxiang Wan, Pedro J. Beltran, Franck J. Calzone, David Elashoff, Pinchas Cohen, William J. Aronson

**Affiliations:** 1 Department of Urology, School of Medicine, University of California-Los Angeles, Los Angeles, CA 90095, USA; E-Mail: cogalet@gmail.com; 2 Department of Integrative Biology and Physiology, University of California-Los Angeles, Los Angeles, CA 90095, USA; E-Mail: angray7@gmail.com; 3 Department of Pathology, School of Medicine, University of California-Los Angeles, Los Angeles, CA 90095, USA; E-Mails: jsaid@mednet.ucla.edu (J.W.S.); bcastor@mednet.ucla.edu (B.C.); 4 USC Davis School of Gerontology, Ethel Percy Andrus Gerontology Center, University of Southern California, Los Angeles, CA 90095, USA; E-Mails: junxianw@usc.edu (J.W.); hassy@usc.edu (P.C.); 5 Oncology Research, Amgen Inc., Thousand Oaks, CA 90095, USA; E-Mails: pbeltran@amgen.com (P.J.B); fjcalzone@roadrunner.com (F.J.C.); 6 Statistic Core, School of Medicine, University of California-Los Angeles, Los Angeles, CA 90095, USA; E-Mail: dae@ucla.edu

**Keywords:** prostate cancer, calorie restriction, IGF-1R blockade

## Abstract

Calorie restriction (CR) inhibits prostate cancer progression, partially through modulation of the IGF axis. IGF-1 receptor (IGF-1R) blockade reduces prostate cancer xenograft growth. We hypothesized that combining calorie restriction with IGF-1R blockade would have an additive effect on prostate cancer growth. Severe combined immunodeficient mice were subcutaneously injected with 22Rv1 cells and randomized to: (1) *Ad libitum* feeding/intraperitoneal saline (Ad-lib); (2) Ad-lib/20 mg/kg twice weekly, intraperitoneal ganitumab [anti-IGF-1R antibody (Ad-lib/Ab)]; (3) 40% calorie restriction/intraperitoneal saline (CR); (4) CR/ intraperitoneal ganitumab, (CR/Ab). CR and ganitumab treatment were initiated one week after tumor injection. Euthanasia occurred 19 days post treatment. Results showed that CR alone decreased final tumor weight, plasma insulin and IGF-1 levels, and increased apoptosis. Ganitumab therapy alone reduced tumor growth but had no effect on final tumor weight. The combination therapy (CR/Ab) further decreased final tumor weight and proliferation, increased apoptosis in comparison to the Ad-lib group, and lowered plasma insulin levels relative to the Ad-lib and Ad-lib/Ab groups. Tumor AKT activation directly correlated with plasma IGF-1 levels. In conclusion, whereas ganitumab therapy modestly affected 22Rv1 tumor growth, combining IGF-1R blockade with calorie restriction resulted in a significant decrease in final tumor weight and improved metabolic profile.

## 1. Introduction

Prostate cancer is the second leading cause of cancer death among men in the United States [1]. Insulin-like growth factor-I (IGF-1) is considered as a factor contributing to prostate cancer risk [2,3]. Epidemiologic studies have reported an association between elevated IGF-1 levels and increased prostate cancer risk [4,5]. IGF-1 also plays a pivotal role in regulating cell proliferation, differentiation, and apoptosis through activation of its receptor, the IGF-1 receptor (IGF-1R) [6].

Based on the role played by IGF-1 in the progression of prostate cancer as well as other malignancies, strong interest exists in developing targeted therapies inhibiting the IGF-1 signaling pathway [3,7]. In a pre-clinical study, the IMC-A12 anti-IGF-1R antibody (ImClone Systems Incorporated, Somerville, NJ, USA) decreased LuCAP prostate cancer xenograft growth [8]. Several biotechnology companies have developed monoclonal antibody therapies against the IGF-1R [9,10]. Clinical trials for the treatment of prostate cancer, both in the neoadjuvant setting and in patients with metastatic, castrate-resistant prostate cancer are ongoing [6]. In a pre-prostatectomy Phase II trial, figitumumab (anti-IGF-1R monoclonal antibody) significantly decreased IGF-1R expression in prostate tissue compared to prostate needle biopsy tissue and decreased PSA values by ≥50% in 31% of patients [11]. Metabolic consequences of IGF-1R-targeted inhibition include elevation in blood glucose and insulin levels via feedback inhibition of the growth hormone/IGF-1 axis [12,13].

Calorie restriction without malnutrition is considered the most potent dietary regimen for suppressing carcinogenesis in mammals [14,15]. A limited number of epidemiological and clinical studies investigated the role of energy intake and/or calorie restriction on prostate cancer. In the Health Professional Follow-up Study, total energy intake was positively associated with increased risk of fatal prostate cancer [16]. A short-term weight loss intervention in obese men resulted in decreased serum IGF-1 levels, increased serum IGFBP-1 levels, and decreased serum-stimulated growth of LNCaP cells in an *ex vivo* bioassay [17]. Calorie restriction inhibits cancer progression through a number of potential mechanisms including reduction in circulating IGF-1 and insulin levels and inhibition of the phosphatidylinositol-3-kinase (PI3K)-Akt pathway [18,19].

We recently published that dietary fat reduction combined with IGF-1R antibody blockade resulted in decreased proliferation in prostate cancer xenografts and a reduction in serum insulin and TNF alpha levels without affecting final tumor weights [13]. Given the lack of effect on final tumor weight and since calorie restriction exerts its anticancer effects, in part, through inhibition of the IGF-1 axis and possibly through reduction of serum insulin levels [18,20], we hypothesized that combining calorie restriction with IGF-1R blocking antibody therapy would cause additive inhibition of prostate cancer progression and potentially offset the insulin-resistance-inducing effects of IGF-1R inhibition.

## 2. Results and Discussion

### 2.1. Results

#### 2.1.1. Reduced 22Rv1 Xenograft Growth in the Calorie Restriction and the Combined Therapy Groups

**Figure 1:**
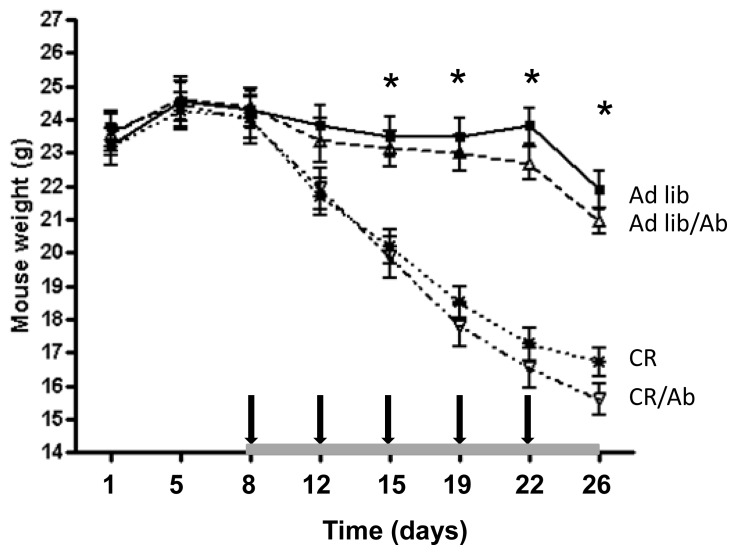
SCID mouse weights. Mice were weighed twice weekly from the day of 22Rv1 cells injection (day 1). Values are expressed as mean ± standard errors (SE). The gray bar on the *x* axis indicates the length of the diet intervention. The arrows indicate the time of saline or ganitumab injections. * indicates significant differences in body weight between mice from Ad-lib groups and the CR groups, *p* < 0.05.

The mice in the Ad-lib and Ad-lib/Ab groups maintained equal calorie intake throughout the experiment with each mouse consuming an average of 10.4 kcal per mouse per day. Mouse weights were also equal between the two groups throughout the study (Figure 1). The mice in the CR and CR/Ab group received 60% of what the Ad-lib and Ad-lib/Ab groups ate throughout the experiment with each mouse receiving 6.2 kcal per mouse per day. Mouse weights were equal between the CR and CR/Ab groups throughout the study. As a result of calorie restriction a significant 27% ± 1.1% weight loss was observed in the CR and CR/Ab groups compared with those in Ad-lib and Ad-lib/Ab groups (Figure 1). Ganitumab did not affect body weight.

**Figure 2:**
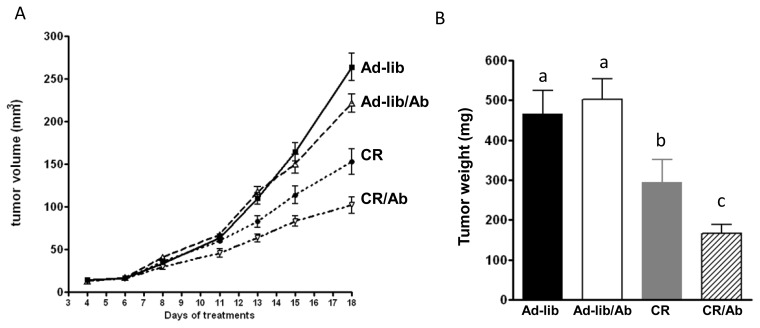
Tumor volumes and weights. (**A**) Tumor volumes: once the tumors became palpable, tumor volume was measured twice weekly. Values are expressed as mean ± SEM; and (**B**) Tumor weights. Values are expressed as mean standard errors (SE). Means with different letters are significantly different from each other (one way analysis of variance). In all cases, statistical significance was considered at *p* < 0.05.

All mice developed tumors. Time of formation of palpable tumor was identical between the groups (Figure 2A). The effect of diet and antibody treatment on tumor growth was assessed using a mixed effect linear model. The treatment effects were identified by interaction with time. Both the antibody therapy and calorie restriction individually affected tumor growth over time (*p* = 0.02 and *p* < 0.001, respectively, Figure 2A), however no significant interaction effect was observed (CR by Ab by time, *p* = 0.13) indicating no synergism between CR and Ab therapy. The absence of synergism was confirmed by two way ANOVA analysis on the final tumor volumes (Figure 2A). No significant difference in final tumor weights was observed between the Ad-lib and Ad-lib/Ab group (*p* = 0.4). Tumor weight was significantly lower in the CR group compared with the Ad-lib groups (*p* < 0.001). Tumor weight in the CR/Ab group was significantly lower (*p* < 0.05) than the other three groups (166 ± 23 mg *vs.* Ad-lib: 467 ± 58 mg, Ad-lib/Ab: 502 ± 52 mg and CR: 295 ± 56 mg) however the interaction effect was not significant (*p* = 0.1; Figure 2B) confirming the absence of synergism between Ab and CR therapy.

#### 2.1.2. Changes in the IGF Axis in Response to the IGF-1R Blocking Therapy and Calorie Restriction

**Figure 3:**
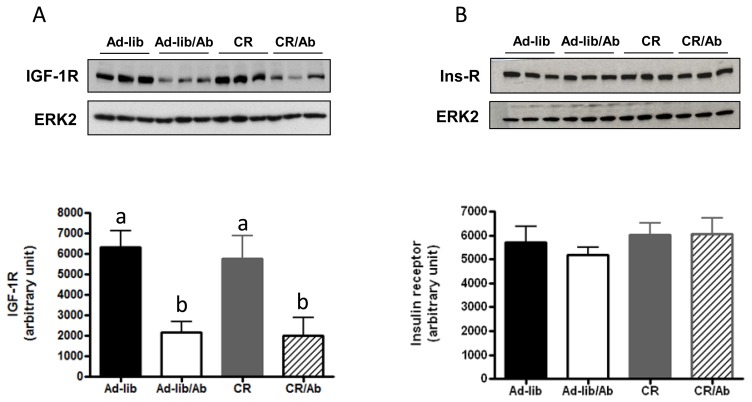
Effect of the intervention on IGF-1 and Insulin receptors expression in 22Rv1 xenografts. (**A**) IGF-1 receptor expression; (**B**) Insulin receptor expression. In (**A**) and (**B**), total ERK2 was used as a loading control. The Western blots are representative of one experiment (*n* = 3 animals per group). The western blots were done on a total of 6 animals per group. Densitometric analysis is presented in the bar graphs for both IGF-1R and the insulin receptor. Means with different letters are significantly different from each other (*p* < 0.05, one way analysis of variance).

Ganitumab induced significant reduction in xenografts’ IGF-1R levels as measured by western blot analysis (Figure 3A), no change in insulin receptor levels was observed (Figure 3B).

**Figure 4:**
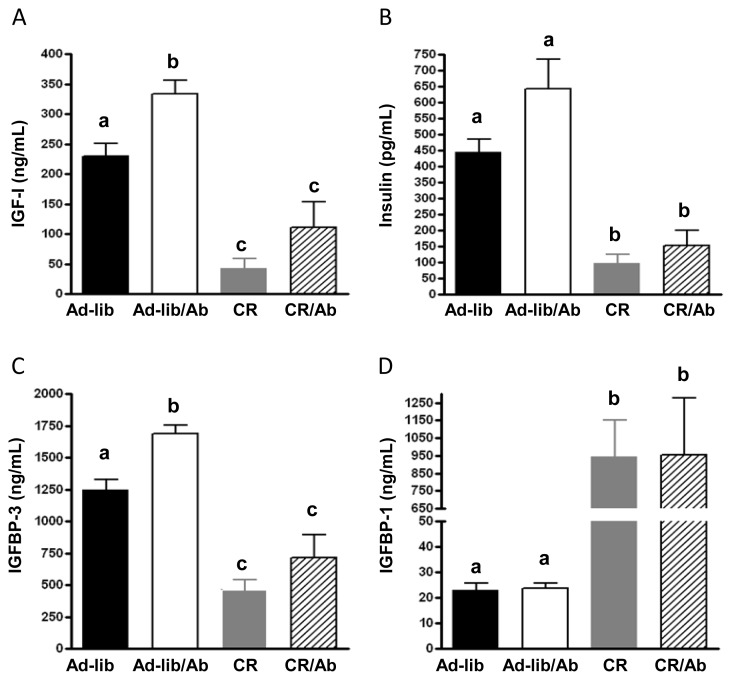
Effect of the intervention on the IGF axis. Fasting plasma concentration of (**A**) IGF-1; (**B**) Insulin I; (**C**) GFBP-3; and (**D**) IGFBP-1 from SCID mice on the different therapy regimen. Plasma IGF-1, IGFBP-1, IGFBP-3 and insulin levels were assessed using ELISA in 6 to 8 animals per group. Values are means ± standard errors (SE). Means with different letters are significantly different from each other (*p* < 0.05, one way analysis of variance).

Plasma IGF-1 and IGFBP-3 levels were significantly elevated in the Ad-lib/Ab group relative to the Ad-lib control group (Figure 4A,C) and significantly lower in the CR and CR/Ab groups relative to the Ad-lib and Ad-lib/Ab groups (Figure 4A,C). A trend for higher plasma insulin levels in the Ad-lib/Ab group compared to the Ad-lib group (*p* = 0.07, Figure 4B) was observed. Insulin levels were significantly decreased in the CR and CR/Ab groups compared to the Ad-lib and Ad-lib/Ab groups (Figure 4B). While insulin levels were higher in the groups receiving ganitumab, the difference was not statistically significant (*p* = 0.3). Plasma IGFBP-1 levels in the CR and CR/Ab groups were significantly higher than in the Ad-lib and Ad-lib/Ab groups (Figure 4D). Two way ANOVA analysis indicated no significant interaction between calorie restriction and ganitumab for IGF-1 (*p* = 0.5), insulin (*p* = 0.3), IGFBP-3 (*p* = 0.4) or IGFBP-1 (*p* = 1). TNF alpha circulating levels were not affected by the different therapies (data not shown).

#### 2.1.3. Effect of Calorie Restriction and IGF-1R Blocking Therapy on 22Rv1 Xenograft AKT Activation, Apoptosis and Proliferation

**Figure 5:**
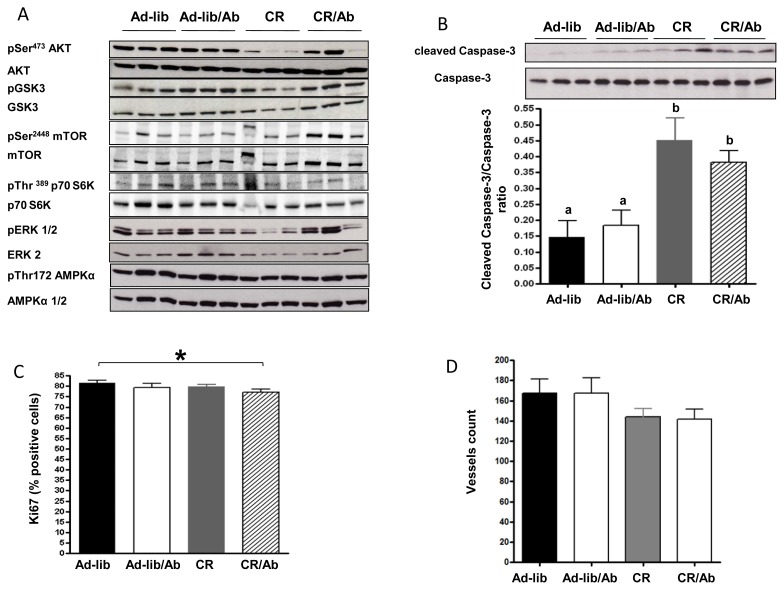
Effect of the different therapies on Akt pathway activation, apoptosis and proliferation. (**A**) Activation of the Akt pathway was assessed by western blotting on xenograft tissue lysate from 6 animals for each group. The Western blots are representative of one experiment (*n* = 3 animals per group); (**B**) Apoptosis was measured by western blotting for cleaved Caspase-3 and toal caspase 3 on xenograft tissue lysate from 6 animals for each group. The Western blots are representative of one experiment (*n* = 3 animals per group). Apoptosis index is measured as a ratio of cleaved-Caspase 3/total Caspase 3. Values are means ± standard errors (SE). Means with different letters are significantly different from each other (*p* < 0.05, one way analysis of variance); (**C**) Proliferation was assessed by Ki67 immmunostaining. 200 cells were manually counted by a blinded pathologist, the Ki67 positive cells were expressed as a percentage of total cells. *: indicate a significant difference *p* < 0.05, Student’s t-test); and (**D**) Angiogenesis was assessed by PECAM/CD31 immunostaining. The number of vessels was counted in five 20× fields for each stained slide.

As shown in Figure 5A, ganitumab treatment alone (Ad-lib/Ab) did not affect AKT activation. Compared with the Ad-lib group, calorie restriction decreased Akt activation in 22Rv1 xenografts from 4 of the 6 animals tested in the CR group, while in the combination therapy (CR/Ab) group Akt activation was lower in 2 of the 6 animals tested. No interaction between ganitumab and calorie restriction was observed on AKT activation using two way ANOVA (*p* = 0.7). Across all treatment groups, Akt activation was positively correlated to plasma IGF-1 levels (*R* = 0.44, *p* < 0.05). No effect of calorie restriction or ganitumab, alone or in combination was observed on GSK3 activation (*p* = 0.9, *p* = 0.1 and *p* = 0.7, respectively), mTOR activation, p70S6Kinase, ERK activation (*p* = 0.3, *p* = 0.2 and *p* = 0.7 respectively) or on AMPK activation (*p* = 0.7, *p* = 0.5 and *p* = 0.7 respectively). To assess the effect of the different therapies on apoptosis, cleaved Caspase-3 was measured in 22Rv1 xenografts by western blotting. Tumors from the Ad-lib and Ad-lib/Ab group had a low mean apoptosis index (cleaved Caspase-3/total Caspase-3 ratio). Calorie restriction, regardless of ganitumab treatment, induced a significant increase in apoptosis (Figure 5B), however no effect of ganitumab on apoptosis was observed (*p* = 0.7).

Two way ANOVA showed no interaction between calorie restriction and ganitumab treatment on apoptosis (*p* = 0.3). Caspase cleavage was inversely correlated to circulating IGF-1 levels across all treatment groups (*R* = −0.5, *p* < 0.05). Xenografts from the CR/Ab group presented a significant 5% decrease in proliferation as measured by Ki67 immunostaining relative to the Ad-lib group (Figure 5C, *p* = 0.04). No significant change in angiogenesis as measured by PECAM/CD31 immunostaining was noted (Figure 5D).

### 2.2. Discussion

Both IGF-1R blocking therapy and calorie restriction were previously found to inhibit prostate cancer xenograft growth [8,18–20]. The present study was designed to investigate whether combining calorie restriction with IGF-1R blocking antibody therapy would cause additive inhibition of prostate cancer progression. IGF-1 receptor blocking therapy alone reduced tumor volume but not tumor weight at the time of euthanasia. Calorie restriction alone (CR) resulted in a significant decrease in tumor growth. The combination therapy (CR/Ab) induced a significant decrease in tumor weights compared to the 3 other groups (Ad-lib, Ab and CR). The effects of calorie restriction regardless of IGF-1 receptor blockade on tumor growth were accompanied with an increase in apoptosis. In addition to affecting apoptosis, the combination therapy also resulted in a decrease in proliferation.

IGF-1R blocking therapy using a different antibody (IMC A12) was previously found to inhibit prostate cancer xenograft progression in LuCaP 35 xenografts *in vivo* [8]. As observed in a previous study [13], ganitumab treatment alone did not affect tumor weight, proliferation, or apoptosis. Ganitumab treatment resulted in down regulation of total IGF-1R in the xenografts; but no significant effect on the activation of Akt or ERK downstream signaling effectors of the IGF-1R was observed. In a recently completed neoadjuvant trial, IGF-1R inhibition decreased expression of IGF-1R in prostatectomy tissue but no effect on phospho-AKT and phospho-p44/42 MAPK was observed [11]. IGF-1R inhibition can induce a resistance mechanism via other growth factor receptors in various cancer cell lines [21,22]. Although we did not explore these mechanisms in our model, the cross-talk between IGF-1R and other activators of Akt may have contributed to our findings. IGF-1R blocking therapy resulted in an increase in plasma levels of IGF-1 and insulin. This metabolic side effect of IGF-1R blocking therapy has been previously observed in pre-clinical and clinical trials [12,13].

Calorie restriction (CR) is a well-established dietary intervention for preventing cancer and increasing lifespan in mammals [14], and is known to reduce prostate tumor growth, angiogenesis, AKT activation and circulating IGF-1 levels in xenograft and transgenic mouse models of prostate cancer [18,19]. Consistent with the literature, we observed a reduction in xenograft growth and an increase in apoptosis accompanied by a significant decrease in circulating IGF-1 and insulin levels in the calorie restricted group. While calorie restriction alone has previously been shown to induce a decrease in proliferation in prostate carcinoma in the Hi-myc transgenic mouse model which has a fully functional immune system [18], we did not observe any effect of calorie restriction alone on proliferation of 22RV1 xenografts in severe combined immunodeficient (SCID) mice, an immunocompromised mouse model. Calorie restriction also had no effect on proliferation in a prior study utilizing a LnCAP xenograft model [19]. One explanation for this discrepancy may be the use of SCID mice. While genetically modified mouse models retain a fully functional immune system, SCID mice present a severely impaired immune system with no differentiation of both lymphocytes T and B [23]. Blando *et al.* reported that 30% calorie restriction in the Hi-myc transgenic mice induced a significant decrease in inflammatory cytokine genes expression suggesting a potential role for inflammation in tumor progression [18].

Combining calorie restriction with IGF-1R blocking antibody therapy caused inhibition of prostate xenograft growth. However, compared to calorie restriction alone, no additional effects were observed on apoptosis or angiogenesis. To understand the mechanisms underlying the growth inhibitory effect of combination therapy; other pathways were studied (androgen receptor, AMPK/mTOR pathway) but none of them were significantly affected by the treatments alone or in combination. Further studies are required to understand the effect of combination therapy on tumor growth and proliferation.

Combined therapy had a beneficial effect on circulating levels of IGF-1 and insulin compared to the IGF-1R antibody alone as previously observed [13]. IGF-1R antibody therapy has been well tolerated in phase I and phase II clinical trials, with hyperglycemia occurring in approximately 3% to 25% of subjects [11,12]. In the present study, serum insulin levels were increased by 44% in the Ad-lib/Ab group as compared to the Ad-lib group while serum insulin levels in the CR/Ab group were significantly lowered (76% reduction) compared to the Ad-lib/Ab group suggesting that calorie restriction offsets the hyperinsulinemia and related metabolic consequences associated with IGF-1R antibody therapy. Some of the adverse effects of cancer therapy have been proposed to be related to circulating IGF-1, and reduction of IGF-1 levels by fasting or genetic ablation have been shown to reduce morbidity and mortality in mouse models [24], and small human case series [25]. Combining pharmacological and nutritional interventions as described in our study, offers the potential for both limiting the untoward effects of drugs that raise IGF-1 and insulin (such as IGF-1R antibodies) and enhancing their anti-tumor effects.

Calorie restriction (CR) extends life span and retards age-related chronic diseases in a variety of species, including rats, mice, fish, flies, worms, and yeast [26]. While a 40% calorie restriction would be a drastic intervention in humans and would most likely not be sustainable, others have shown the feasibility of lesser degrees of calorie restriction. Fontana et al reported that a long term (4 years) 15% calorie restriction intervention was feasible and resulted in a decrease of plasma growth factors and hormones linked to an increased risk of cancer [27–29]. In the CALERIE Study participants were compliant with a 25% calorie restriction intervention or a 12.5% calorie restriction combined with a 12.5% energy expenditure program [30]. Thus calorie restriction in human trials is feasible yet further prospective trials are warranted to assess the feasibility of calorie restriction in prostate cancer patients.

## 3. Experimental Section

### 3.1. Animal Husbandry and Feeding Protocol

The experiments described herein were approved by the UCLA Chancellor’s Animal Research Committee, and animals were cared for in accordance with institutional guidelines. Sixty-five male CB17 beige severe combined immunodeficient (SCID) mice (8 weeks old) were obtained from the UCLA Department of Laboratory Animal Medicine facility (accredited by the American Association for Accreditation of Laboratory Animal Care). The mice were housed one per cage. The cages were kept in a sterile and pathogen-free facility and sterile techniques were used as previously described [13]. The diets were prepared and sterilized (by irradiation) by DYETS, Inc. (Bethlehem, PA, USA). The *ad-libitum* diet contained 20% calories from fat. The calorie restriction group diet contained 20% calories from fat and was supplemented with 40% more vitamin and minerals so that all groups received the same amount of vitamins and minerals (Table 1). Food intake and animal health was monitored daily.

### 3.2. 22Rv1 Cell Line

22Rv1 cells were cultured as previously described [13]. IGF-1 receptor blockade was previously shown to have antiproliferative and pro-apoptotic effects on 22RV1 cells *in vitro* [13].

### 3.3. Experimental Design

All mice were fed the *ad libitum* diet for two weeks prior to being injected in the lateral flank with 1 × 10^5^ 22Rv1 cells in 0.2 mL of a solution containing the cells in 0.1 mL of RPMI 1640 media and 0.1 mL of matrigel (BD Biosciences, Bedford, MA, USA). Mice continued consuming the *ad libitum* diet for a week following injection, at which point they were randomized into four groups. Two mice did not present palpable tumors and were euthanized. The remaining 63 mice were randomized as follow: (1) *Ad libitum* with intraperitoneal saline (Ad-lib), *n* = 16; (2) *Ad-libitum* with intraperitoneal IGF-1R blocking antibody, ganitumab (AMG 479) (Ad-lib/Ab,), *n* = 16; (3) 40% Calorie restriction with intraperitoneal saline (CR), *n* = 16; and (4) 40% calorie restriction with intraperitoneal ganitumab (CR/Ab), *n* = 15. The calorie restricted groups were fed 60% of what the Ad-lib group ate. Ganitumab antibody was administered at a dose of 20 mg/kg similar to what is used in human [31]. Ganitumab and intraperitoneal saline were administered twice weekly. Mice were weighed twice weekly and tumor dimensions were measured three times a week using a caliper. Tumor volumes were calculated using the formula previously described: length × width × height × 0.5236 [13].

### 3.4. Plasma and Tumor Collection

Plasma and tumor were collected as previously described [13].

### 3.5. Plasma Studies

The levels of murine IGF-1, IGFBP-1 and IGFBP-3 were measured using in-house mouse-specific ELISAs as previously described [13]. Plasma insulin levels were measured using an insulin (mouse) ultrasensitive ELISA (Alpco, Salem, NH, USA). Plasma TNF-alpha levels were measured using commercially available ELISA (BD Bioscience, San Jose, CA, USA).

### 3.6. Immunohistochemistry

Four micrometer formalin-fixed tumor sections embedded in paraffin were stained with H & E, Ki67 and Pecam1/CD31 as previously described [13].

### 3.7. Western Blot Analysis

Western blots were run under reducing conditions as previously described [13]. IGF-1R, Insulin receptor, p-AKT, AKT, IRS-1 and 2, Caspase-3, pAMPK, AMPK, p-mTOR, mTOR, p-GSK3, GSK3, p-p70S6K, p70S6 and androgen receptor antibodies were from Cell Signaling Technology (Danvers, MA, USA). Anti-p-ERK1/2 and ERK 2 were purchased from Santa Cruz Biotechnology Inc. (Dallas, TX, USA).

### 3.8. Statistical Analysis

Normal distribution was verified using the D’Agostino & Pearson omnibus normality test. Statistical analyses were conducted using unpaired t tests, one-way analysis of variance (ANOVA) followed by a Tukey post-hoc test or two way ANOVA followed by a Bonferroni multiple comparison post-hoc test. The tumor volume curves were evaluated using mixed effects linear models. These models contained terms for the time by treatment (Ab, CR) interactions as well as the three way (time by Ab by CR) interaction effect. Correlation between weight loss and tumor weights, IGF-1, IGFBP-3, IGFBP-1 Caspase-3 and correlations between IGF-1 and Akt or Caspase-3 were analyzed using the Pearson correlation coefficient. Statistical significance was considered at *p* < 0.05. Analyses were conducted using GraphPad Prism5 (GraphPad Software Inc., San Diego, CA, USA) and R version 2.13.2 [32].

## 4. Conclusions

In summary, calorie restriction alone and in combination with IGF-1R antibody blockade resulted in decreased growth of prostate cancer xenografts. Further preclinical and clinical trials are warranted to evaluate combining calorie restriction with IGF-1 receptor blockade to enhance the efficacy and offset the metabolic consequences of antibody therapy directed against the IGF-1 receptor.

## Figures and Tables

**Table 1:** Ingredients of experimental diets.

Ingredient	kcal/g	*Ad libitum* diet		Calorie restriction diet	
	
		g/kg	kcal/kg	g/kg	kcal/kg
Casein	3.6	200	716	200	716
L-Cystine	4	3	12	3	12
Sucrose	4	84	336	84	336
Cornstarch	3.6	397.5	1431	378.5	1363
Dyetrose	3.8	132	501.6	132	501.6
Corn Oil	9	86	774	86	774
Cellulose	0	50	0	50	0
Mineral Mix #210025	0.9	35	30.8	49	43.1
Vitamin Mix #310025	3.9	10	38.7	14	54.2
Choline Bitartrate	0	2.5	0	3.5	0
Total		1000	3840.1	1000	3799.9
